# Quercetin alleviates rifampicin-induced hepatocyte injury by modulating the Hippo-YAP signaling pathway

**DOI:** 10.3389/fmed.2025.1707248

**Published:** 2025-11-18

**Authors:** Weiwei Liu, Hui Li, Weimin Lu

**Affiliations:** The First School of Clinical Medicine, Nanjing University of Chinese Medicine, Nanjing, China

**Keywords:** quercetin, Hippo–YAP, drug-induced liver injury, apoptosis, rifampicin

## Abstract

**Objective:**

To investigate the protective effects of Quercetin, an antioxidant and anti-apoptotic flavonoid, against rifampicin (RFP)-induced hepatocyte injury via the Hippo-YAP signaling pathway.

**Methods:**

A rifampicin-induced hepatocyte injury model was established using HepaRG cells. HepaRG cells were divided into Control, RFP Model, Quercetin (15 μM), and Verteporfin (YAP inhibitor) groups. Cell viability was assessed by the CCK-8 assay, apoptosis by Annexin V/PI staining, and mitochondrial membrane potential (MMP) by TMRE staining. YAP localization was evaluated by immunofluorescence, and the expression of Hippo–YAP and apoptosis-related genes was analyzed by Western blot and qRT-PCR.

**Results:**

Quercetin significantly improved cell viability, reduced apoptosis, and restored MMP in RFP-injured HepaRG cells. RFP activated the Hippo pathway by upregulating MST1 and LATS1, increasing YAP phosphorylation, and promoting apoptosis-related protein expression (Caspase-3, BAX), while downregulating anti-apoptotic BCL-2. Quercetin reversed these effects by inhibiting MST1/LATS1 activation, reducing YAP phosphorylation, and promoting its nuclear translocation. The protective effects were partially attenuated by Verteporfin, indicating Hippo–YAP pathway involvement.

**Conclusion:**

Quercetin alleviates RFP-induced hepatocyte injury by suppressing Hippo pathway kinases MST1 and LATS1, reducing YAP phosphorylation, and enhancing YAP nuclear translocation, thereby improving MMP and inhibiting apoptosis.

## 1 Introduction

The liver, positioned between the absorption and systemic circulation systems, plays a vital role in drug metabolism and elimination, making it highly susceptible to drug-induced toxicity. Drug-induced liver injury (DILI), characterized by damage to hepatocytes and other liver cells, poses a significant challenge in hepatology (1). Worldwide, DILI is recognized as a major cause of acute liver failure (2, 3). Rifampicin (RFP), a commonly used first-line drug for tuberculosis, is known to induce serious liver toxicity, including conditions such as cholestasis and hyperbilirubinemia (4–6). Despite its efficacy, long-term RFP use often results in hepatotoxicity characterized by hepatocyte apoptosis, oxidative stress, and mitochondrial dysfunction (7, 8). RFP can disrupt hepatic lipid metabolism by upregulating genes involved in fatty acid synthesis and uptake, including CD36, and by activating the pregnane × receptor, which subsequently increases the expression of peroxisome proliferator-activated receptor-γ and its downstream proteins such as perilipin-2, leading to hepatic steatosis and liver dysfunction (8). Current therapeutic approaches, such as hepatoprotective agents and antioxidants, have shown limited efficacy and are often associated with adverse effects, emphasizing the need for safer and more effective interventions (9).

Flavonoids, a diverse group of plant-derived polyphenols, have attracted growing attention for their hepatoprotective potential. Numerous studies have shown that flavonoids exert antioxidant, anti-inflammatory, and anti-apoptotic effects in models of DILI, helping to preserve mitochondrial function and attenuate oxidative stress (10, 11). Among these compounds, Quercetin—a dietary flavonoid abundant in onions, apples, and tea—has been extensively studied for its broad biological activities, including antioxidant, anticarcinogenic, anti-inflammatory, anti-diabetic, and antimicrobial properties (12–14). *In vitro* and *in vivo* studies have demonstrated that Quercetin and its metabolites can inhibit xanthine oxidoreductase, regulate blood uric acid levels, and modulate mitochondrial biogenesis, membrane potential, and redox balance, ultimately influencing mitochondria-dependent apoptosis (15–17). Quercetin is recognized as a phytochemical that can modulate pathways associated with mitochondrial biogenesis, mitochondrial membrane potential, oxidative respiration and ATP anabolism, intra-mitochondrial redox status, and subsequently, mitochondria-induced apoptosis (18). The anti-cancer effects of Quercetin include its ability to promote the loss of cell viability, apoptosis and autophagy through the modulation of PI3K/Akt/mTOR, Wnt/β-catenin, and MAPK/ERK1/2 pathways (19).

Recent evidence highlights the Hippo–YAP signaling pathway as a central regulator of hepatocyte proliferation, apoptosis, and tissue regeneration (20, 21). Within this pathway, Yes-associated protein (YAP) acts as the principal downstream effector. When phosphorylated by the MST/LATS kinase module in cooperation with MOB1, YAP is sequestered in the cytoplasm or degraded via β-Trcp-mediated ubiquitination. Conversely, when the Hippo pathway is inactive, unphosphorylated YAP translocates into the nucleus, binds to TEAD transcription factors (TEAD1–4), and activates genes such as CTGF, Cyr61, Ccnd1, E2f1, and Birc5, which are crucial for cell proliferation and tissue repair (22–24). The activity of the Hippo–YAP signaling pathway changes dynamically with the liver's regenerative capacity. Elevated YAP expression has been found to promote liver regeneration following partial hepatectomy or toxic damage, while suppression of YAP produces the opposite effect (25, 26). The dynamic activity of this pathway has been shown to correlate with liver regenerative potential—with YAP activation enhancing regeneration following partial hepatectomy or toxic injury, and YAP inhibition exerting the opposite effect.

Given Quercetin's regulatory influence on multiple intracellular signaling networks and its recognized hepatoprotective capacity, it is plausible that Quercetin may exert part of its protective action through modulation of the Hippo–YAP pathway. Nevertheless, whether and how Quercetin influences YAP activation in the context of RIF-induced hepatocyte injury has not yet been elucidated. Therefore, this study aims to investigate the role of the Hippo–YAP signaling axis in Quercetin-mediated hepatoprotection, providing new insights into its molecular mechanism against drug-induced liver damage.

## 2 Materials and methods

### 2.1 Cell culture and grouping

Human hepatic progenitor HepaRG cells (IM-H415) were purchased from Xiamen Yimo Biotechnology Co., Ltd. These cells possess the ability to differentiate into hepatocyte-like and biliary-like cells, making them a widely accepted *in vitro* model for human hepatocyte metabolism and drug-induced liver injury studies. The cells were cultured in high-glucose DMEM medium (Gibco, USA) supplemented with 10% fetal bovine serum (ExCell Bio, China) and 1% penicillin-streptomycin solution (Beyotime, China). The cells were maintained in an incubator (CI-150C, Suzhou Jiemei Electronic Co., Ltd.) at 37 °C with 95% humidity and 5% CO_2_. When cell density reached more than 80%, the cells were digested with 0.25% trypsin (Gibco, USA) for 1 min, neutralized with complete medium, resuspended, and subcultured.

The HepaRG cells were divided into four groups for the experiments: (1) Control group: Cells were cultured in regular DMEM medium without RFP or Quercetin; (2) Model group: Cells were exposed to 25 μm RFP for 48 h to induce hepatocellular injury (27); (3) Quercetin group: Cells were treated with 25 μm RFP in combination with 15 μm Quercetin for 48 h in light-protected conditions; (4) Verteporfin group: Cells were treated with 25 μM RFP in combination with 15 μM Quercetin and 2 μM YAP inhibitor Verteporfin for 48 h in light-protected conditions (28). By comparing the effects of Quercetin alone and in combination with Verteporfin, we were able to determine whether YAP activation contributes to Quercetin's anti-apoptotic, thus clarifying the pathway's mechanistic role.

### 2.2 Cell viability assay

The cell viability of HepaRG cells was evaluated using the Cell Counting Kit-8 (CCK-8, Beyotime, China). Cells were seeded into 96-well plates at a density of 2,000 cells per well and incubated at 37 °C with 5% CO_2_ for 6 h to allow attachment. Cells were then treated with 25 μm RFP for 48 h to establish a liver injury model, followed by exposure to Quercetin at varying concentrations (2.5, 5, 10, 15, and 20 μm) for an additional 48 h. 10 μl of CCK-8 reagent was added to each well, and absorbance at 450 nm was measured using a microplate reader (Thermo Scientific, Multiskan FC). The optimal Quercetin concentration (15 μm), determined from preliminary experiments, was used in subsequent assays for the experimental groups.

### 2.3 Flow cytometry

Logarithmic-phase HepaRG cells were seeded into 6-well plates at a density of 1 × 106 cells per well and cultured at 37 °C with 5% CO_2_. Once cells adhered, treatments were applied according to the experimental grouping. Following treatment, cells were harvested, washed twice with PBS, and resuspended in 1× Annexin V Binding Buffer at 1 × 106 cells/ml. A 100 μl aliquot was incubated with 5 μl Annexin V-FITC and 5 μl PI in the dark at room temperature for 15 min, followed by the addition of 400 μl Binding Buffer. Samples were analyzed within 1 h using a flow cytometer (Attune NxT, Thermo Fisher, USA). Early (Annexin V^+^/PI^−^) and late (Annexin V^+^/PI^+^) apoptotic cells were quantified using the instrument's analysis software.

### 2.4 Western blot

After treatment, HepaRG cells were lysed in RIPA buffer containing PMSF (Beyotime, China). Lysates were centrifuged at 12,000 × g for 15 min at 4 °C, and the supernatants were collected. Protein concentrations were determined using a BCA assay kit (NCM Biotech, China). Equal amounts of protein were mixed with 5 × SDS loading buffer, boiled for 5 min, separated on 10% SDS-PAGE gels, and transferred onto PVDF membranes (Sigma-Aldrich, USA). Membranes were blocked with 5% skim milk in TBST for 1 h at room temperature, then incubated overnight at 4 °C with primary antibodies against Phospho-YAP (Ser127) (#13008, CST, 1:1000), YAP (#4912, CST, 1:1000), LATS1 (#3477, CST, 1:1000), MST1 (#3682, CST, 1:1000), Caspase-3 (#ab32351, Abcam, 1:1000; recognizes both the pro-form and the p17 cleaved form of human Caspase-3), Bcl-2 (#15071, CST, 1:1000), Bax (#2772, CST, 1:1000), and GAPDH (#2118, CST, 1:1000). After three TBST washes (10 min each), membranes were incubated with HRP-conjugated secondary antibodies (anti-rabbit or anti-mouse, Bioss, China; 1:20,000) for 1 h at room temperature. Protein bands were visualized using an ECL kit (NCM Biotech, China) and imaged with a chemiluminescence detection system (JP-K6000, Jiapeng, China). Band intensities were quantified using ImageJ, and protein expression levels were normalized to GAPDH.

### 2.5 Quantitative real-time PCR (qRT-PCR)

Total RNA was extracted from HepaRG cells using the Cell/Tissue Total RNA Isolation Kit V2 (RC112, Vazyme, China) according to the manufacturer's instructions. The concentration and purity of the extracted RNA were determined using a Nano-600 micro-spectrophotometer (Shanghai Jiapeng Technology, China). Subsequently, 1 μg of total RNA was reverse-transcribed into cDNA using the HiScript III 1st Strand cDNA Synthesis Kit (R312, Vazyme, China). Quantitative real-time PCR (qRT-PCR) was performed with Taq Pro Universal SYBR qPCR Master Mix (Q712, Vazyme, China) using 2 μL of cDNA template per 20 μl reaction. Amplification was carried out on a CFX96 Touch Real-Time PCR System (Bio-Rad, USA) under the following thermal cycling conditions: an initial denaturation at 95 °C for 30 s, followed by 40 cycles of 95 °C for 10 s and 60 °C for 10 s. A melting curve analysis was conducted at the end of the amplification program (95 °C for 15 s, 65 °C for 60 s). Relative mRNA expression levels were calculated using the 2^−ΔΔCt^ method, with GAPDH serving as the internal reference. The primer sequences used for qRT-PCR are listed in Table 1.

**Table 1:** Primer sequences.

**Genes**	**Primer sequences (5^′^3^′^)**
YAP	Forward: CCCTCGTTTTGCCATGAACC
	Reverse: GTTGCTGCTGGTTGGAGTTG
LATS1	Forward: ATCAGCAGCGTCTACATCGT
	Reverse: AAACACCAAGCAAACAGATGAT
MST1	Forward: TGTGCACTGAGGGACTGTTG
	Reverse: AGAGACACGCGTGAAGACAG
Caspase-3	Forward: TGCTATTGTGAGGCGGTTGT
	Reverse: TCACGGCCTGGGATTTCAAG
Bcl-2	Forward: GAACTGGGGGAGGATTGTGG
	Reverse: CCGTACAGTTCCACAAAGGC
Bax	Forward: GAGCAGCCCAGAGGCG
	Reverse: TGAGACACTCGCTCAGCTTC
GAPDH	Forward: AATGGGCAGCCGTTAGGAAA
	Reverse: GCGCCCAATACGACCAAATC

### 2.6 Mitochondrial membrane potential

Mitochondrial membrane potential (MMP) was evaluated using tetramethylrhodamine ethyl ester (TMRE) staining (C2001S, Beyotime, China). After treatment according to the experimental groups, cells were washed once with PBS and incubated with 1 ml TMRE working solution at 37 °C with 5% CO_2_ for 30 min. Following incubation, cells were washed twice with pre-warmed PBS and replenished with 2 ml of complete medium. TMRE fluorescence was observed using a fluorescence microscope (BZ-H4XD, Keyence, Japan) at excitation/emission wavelengths of 550/575 nm. Fluorescence intensity was semi-quantitatively analyzed using ImageJ, and MMP changes were expressed as relative TMRE fluorescence intensity.

### 2.7 Immunofluorescence (IF)

After treatment according to the experimental groups, cells were washed with PBS and fixed with 4% paraformaldehyde (Sigma-Aldrich, USA) for 10 min at room temperature. Cells were then permeabilized with 0.25% Triton X-100 (Beyotime, China) for 10 min, washed, and blocked with 10% goat serum (Biosharp, China) for 1 h. The samples were incubated overnight at 4 °C with a primary antibody directly conjugated to Alexa Fluor^®^ 488 (Rabbit Recombinant Monoclonal YAP1 antibody, ab225441, Abcam; 1:200), which specifically binds to human and mouse YAP and is suitable for ICC/IF. No secondary antibody was applied to avoid duplication of fluorophore labeling. After washing, nuclei were counterstained with DAPI (5 μg/ml, Thermo Fisher, USA) for 10 min. Coverslips were mounted with PBS, and fluorescence images were captured using a confocal laser scanning microscope (Leica CTSSP8, Leica Microsystems, Germany).

### 2.8 Statistics analysis

Data were analyzed and visualized using GraphPad Prism 9 (Version 9.5.1) and ImageJ software, with figure assembly performed in Adobe Illustrator (2023). All results are expressed as mean ± standard deviation (mean ± SD). One-way ANOVA followed by Tukey's *post-hoc* test was applied for multiple group comparisons for Type I error. Statistical significance was defined as *P* < 0.05 (^*^P < 0.05, ^**^P < 0.01, ^***^P < 0.001). All experiments were independently repeated at least three times to ensure the reliability and reproducibility of the results.

## 3 Results

### 3.1 Quercetin improved MMP and inhibited RFP-induced apoptosis in HepaRG cells

**Figure 1:**
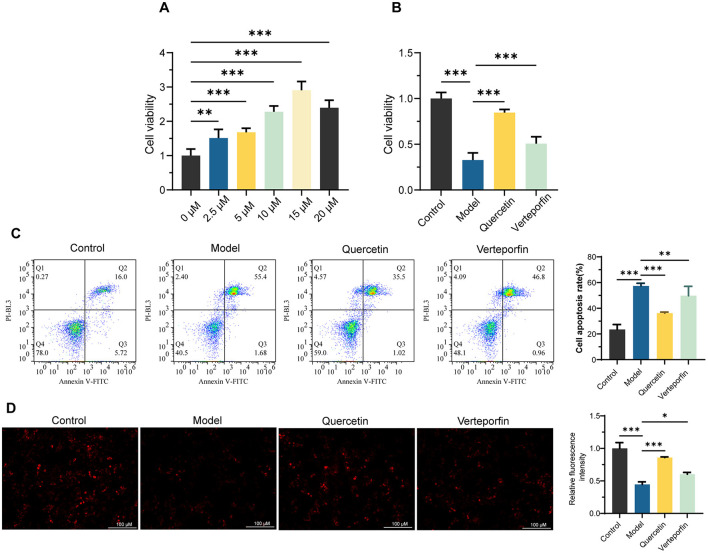
Quercetin improved mitochondrial membrane potential (MMP) and inhibited Rifampicin (RFP)-Induced apoptosis in HepaRG Cells. **(A)** CCK-8 experiment to determine the optimal Quercetin concentration for enhancing HepaRG cell viability (*n* = 6). **(B)** CCK-8 assay to assess the protective effect of Quercetin (15 μm) on cell viability in RFP (25 μm)-treated HepaRG cells. Verteporfin (2 μm) was used to inhibit YAP (*n* = 6). **(C)** Flow cytometry scatter plots and quantification of apoptosis rates of Annexin V/PI staining (*n* = 3). **(D)** The mitochondrial membrane potential was assessed using tetramethylrhodamine ethyl ester (TMRE) and quantification of relative fluorescence intensity (*n* = 3). Scale bars: 100 μm. Data are mean ± SD. *P < 0.05, **P < 0.01, ***P < 0.001. One-way ANOVA followed by Tukey's *post-hoc* test was applied for multiple group comparisons for Type I error.

The CCK-8 assay revealed that Quercetin enhanced HepaRG cell viability in a concentration-dependent manner, reaching its maximum effect at 15 μm, which was selected for subsequent experiments (Figure 1A). Quercetin significantly restored RFP-induced reductions in cell viability (*P* < 0.001), while the addition of Verteporfin slightly weakened this effect but still maintained higher viability than the Model group (*P* < 0.001) (Figure 1B). Flow cytometry analysis demonstrated that Quercetin markedly reduced RFP-induced apoptosis (*P* < 0.001), whereas Verteporfin partially reversed this protection (Figure 1C). Moreover, MMP decreased significantly following RFP treatment but was restored by Quercetin, with Verteporfin again diminishing this recovery effect (Figure 1D). Together, these results indicate that Quercetin alleviates RFP-induced cytotoxicity and mitochondrial dysfunction, effects that are partially dependent on YAP activity.

### 3.2 Quercetin promoted YAP nuclear translocation and alleviated RFP-induced HepaRG cell injury by regulating the hippo-YAP pathway

**Figure 2:**
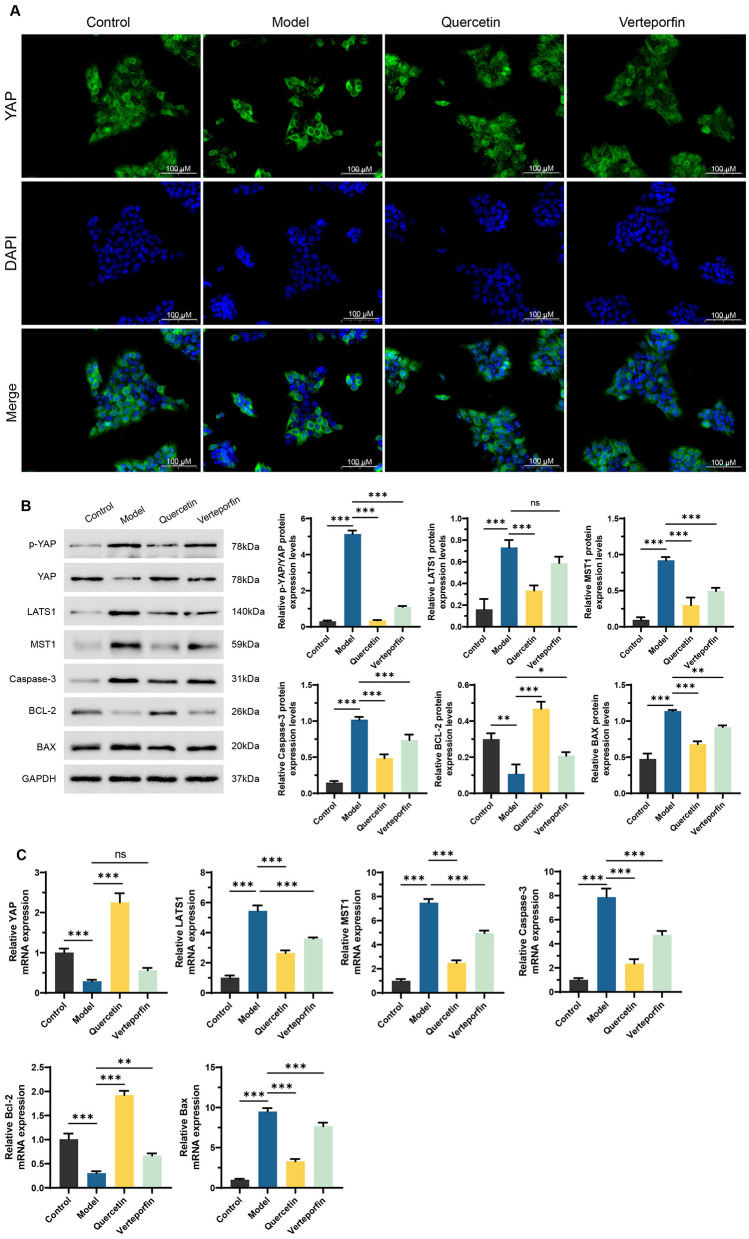
Quercetin alleviates RFP-induced HepaRG cell damage by regulating the Hippo-YAP pathway. **(A)** Immunofluorescence staining for YAP (green) and nuclei (DAPI, blue) was performed on HepaRG cells. Red arrows indicate strong green fluorescence in the cytoplasm, while yellow arrows mark its accumulation in the nuclei. the Scale bars: 100 μm. **(B)** Western Blot analysis of key proteins in the Hippo-YAP pathway and apoptosis-related proteins, including p-YAP, YAP, LATS1, MST1, **total Caspase-3**, BCL-2, and BAX. Quantification of relative protein expression normalized to GAPDH is shown below. **(C)** qRT-PCR analysis of the mRNA expression levels of Hippo-YAP pathway and apoptosis-related genes, including YAP, LATS1, MST1, Caspase-3, BCL-2, and BAX. Verteporfin (2 μm) co-treated with Quercetin (15 μm). NS, no significance. **P < 0.01, ***P < 0.001, *n* = 3. One-way ANOVA followed by Tukey's *post-hoc* test was applied for multiple group comparisons for Type I error.

Immunofluorescence analysis revealed that RFP treatment caused YAP to remain mainly in the cytoplasm (Red arrows), while Quercetin promoted its nuclear translocation (Yellow arrows); co-treatment with Verteporfin reversed this effect (Figure 2A). Western blot results showed that RFP increased p-YAP, LATS1, MST1, total Caspase-3, and BAX levels while decreasing YAP and BCL-2, suggesting activation of the Hippo pathway and induction of apoptotic signaling. Quercetin treatment reversed these trends by reducing p-YAP and pro-apoptosis proteins and elevating YAP and BCL-2 levels, whereas Verteporfin weakened this protective response (Figure 2B). qRT-qPCR analysis confirmed these findings, showing similar transcriptional changes (Figure 2C). Overall, Quercetin mitigated RFP-induced HepaRG cell injury by suppressing YAP phosphorylation, promoting its nuclear translocation, and modulating Hippo-YAP–related apoptotic signaling. However, as only total Caspase-3 was assessed, the apoptotic response should be interpreted cautiously. Future studies incorporating cleaved Caspase-3 or cleaved PARP detection will help strengthen apoptosis validation.

## 4 Discussion

This study reveals the mechanism by which Quercetin alleviates RFP-induced HepaRG cell injury through the activation of the Hippo-YAP signaling pathway. The findings demonstrate that RFP significantly activates the Hippo pathway by promoting YAP phosphorylation, inhibiting its nuclear translocation, and upregulating the expression of pro-apoptotic proteins Caspase-3 and BAX while downregulating the anti-apoptotic protein BCL-2. These changes, along with the disruption of mitochondrial membrane potential, collectively lead to apoptosis. Quercetin, by contrast, reduces YAP phosphorylation, promotes its nuclear translocation, suppresses the overactivation of upstream kinases LATS1 and MST1, and restores cell viability, showcasing its multifaceted protective mechanisms.

Previous studies have established that RFP, a widely used first-line anti-tuberculosis drug, is associated with hepatotoxicity during long-term use, commonly referred to as DILI. The hepatotoxicity manifests as hepatocyte apoptosis, oxidative stress, and mitochondrial dysfunction (4–8). RFP has been shown to induce oxidative stress and activate the mitochondrial apoptotic pathway by triggering Caspase-3 activation, ultimately leading to hepatocyte death (29). The Hippo-YAP signaling pathway, recognized as a critical regulator of cell proliferation, apoptosis, and regeneration, has gained increasing attention for its role in liver diseases (30, 31). For example, overactivation of upstream kinases MST1/2 and LATS1/2 in the Hippo pathway has been reported to suppress YAP nuclear translocation, thereby accelerating apoptosis and tissue injury (32). This study corroborates these findings in a RFP-induced liver injury model. IF staining results showed that RFP treatment led to YAP predominantly localizing in the cytoplasm of HepaRG cells, indicating its impaired nuclear translocation. Western Blot and qRT-PCR results further confirmed that this cytoplasmic localization was associated with a significant increase in YAP phosphorylation levels, along with enhanced expression of upstream kinases MST1 and LATS1. These findings suggest that RFP-induced liver injury is mediated, at least in part, through the dysregulation of the Hippo-YAP signaling pathway, providing a mechanistic foundation for understanding RFP-induced hepatocyte damage.

Quercetin, due to its natural origin and multiple biological properties, is considered a potential hepatoprotective agent (12). In models of metabolic dysfunction-associated steatotic liver disease (MASLD) and acrylamide-induced liver injury, Quercetin has demonstrated significant antioxidant and anti-apoptotic effects (12, 33). Moreover, the role of Quercetin in oxidative stress-related diseases has been extensively studied, with evidence showing that it protects hepatocyte function by inhibiting the generation of reactive oxygen species (ROS) (34, 35). However, most studies have focused on the antioxidant or anti-inflammatory effects of Quercetin. In this study, Western Blot and qRT-PCR analyses revealed that Quercetin significantly reduced YAP phosphorylation levels by suppressing the expression of MST1 and LATS1. Immunofluorescence demonstrated that Quercetin promoted YAP nuclear translocation, restoring its functional activity in the nucleus. Furthermore, Quercetin was shown to restore the expression of the anti-apoptotic gene BCL-2 while suppressing the expression of pro-apoptotic genes BAX and Caspase-3, thereby alleviating apoptosis in RFP-induced HepaRG cells. Annexin V/PI double staining via flow cytometry confirmed that RFP significantly induced both early and late apoptosis in HepaRG cells, while Quercetin markedly inhibited apoptosis. Notably, the addition of the YAP inhibitor Verteporfin resulted in a significant increase in apoptosis rates, highlighting the critical role of YAP in Quercetin-mediated anti-apoptotic effects. These findings support the hypothesis that Quercetin inhibits apoptotic signaling cascades by suppressing Hippo pathway activity, thereby enabling YAP nuclear translocation and downstream survival signals, suggesting that YAP nuclear translocation may directly influence cell survival by regulating the expression of apoptosis-related genes.

Mitochondrial dysfunction resulting from stress is a key mechanism underlying DILI (36, 37). In this study, TMRE staining revealed that RFP significantly decreased mitochondrial membrane potential, while Quercetin effectively restored this parameter. This suggests that Quercetin may stabilize mitochondrial function by reducing ROS-induced mitochondrial pathway activation. Additionally, the inhibitory effect of Quercetin on LATS1/MST1 may indirectly contribute to the recovery of mitochondrial function. Future studies could further explore the interaction between the Hippo-YAP pathway and mitochondrial stability to clarify these mechanisms.

This study provides novel insights into the mechanisms by which Quercetin mitigates rifampicin-induced hepatocyte injury, specifically through activation of the Hippo-YAP signaling pathway. While prior studies have established Quercetin's hepatoprotective effects and the role of YAP in liver regeneration, this work is the first to link Quercetin's anti-apoptotic and mitochondrial protective actions to Hippo-YAP pathway modulation in the context of rifampicin toxicity. The experimental design, combining pharmacological inhibition of YAP (Verteporfin) with functional assays, strengthens the mechanistic evidence for Quercetin's therapeutic potential. Clinically, these findings suggest that Quercetin, as a natural and low-toxicity compound, could serve as an adjunct therapy to mitigate rifampicin-associated liver injury during tuberculosis treatment.

Despite revealing the significant role of Quercetin in mitigating DILI through the regulation of the Hippo-YAP pathway, this study has certain limitations. First, while the HepaRG cell model closely mimics human hepatocyte physiology, the findings require validation *in vivo* systems to confirm Quercetin's efficacy and safety. Second, the Hippo-YAP pathway's complexity and crosstalk with other signaling pathways, such as Wnt or PI3K/Akt, were not fully explored. Third, this study relied on pharmacological YAP inhibition using Verteporfin, which may involve off-target effects. Future work should include a Verteporfin-only control group and adopt genetic approaches (e.g., siRNA/shRNA-mediated YAP silencing) to enhance mechanistic specificity. Moreover, employing YAP agonists such as XMU-MP-1 could provide bidirectional validation of the Hippo-YAP pathway's role in Quercetin's hepatoprotective effects. Fourth, growing evidence suggests that Quercetin also influences autophagic processes, which are closely linked to hepatic protection and cellular stress responses (15, 33, 34). Exploring the interaction between Quercetin-induced autophagy and the Hippo-YAP pathway may provide valuable insights into the broader regulatory mechanisms underlying its hepatoprotective effects and represent a promising direction for future research. Additionally, it remains unclear whether Hippo-YAP activation is a specific response to rifampicin-induced injury or a general hepatocyte stress mechanism, the specific mechanism by which Quercetin regulates this pathway still requires further experimental verification. Finally, as a multi-target compound, Quercetin's effects may vary in complex pathological contexts, necessitating advanced tools like single-cell sequencing to dissect its context-dependent actions.

## 5 Conclusion

This study provides the first evidence that Quercetin alleviates RFP-induced HepaRG cell injury by modulating the Hippo-YAP signaling pathway. Specifically, Quercetin suppressed the activation of the upstream kinases MST1 and LATS1, reduced YAP phosphorylation, and promoted its nuclear translocation, thereby attenuating apoptosis and improve mitochondrial membrane potential. These findings offer novel theoretical insights into the hepatoprotective mechanisms of Quercetin and suggest promising strategies for developing new treatments for DILI. Future research should incorporate *in vivo* studies to further investigate the interactions between Quercetin and upstream and downstream factors of the Hippo-YAP pathway, as well as its crosstalk with other signaling pathways, to establish a solid foundation for clinical applications.

## Data Availability

The original contributions presented in the study are included in the article/supplementary material, further inquiries can be directed to the corresponding authors.
